# Preliminary Evaluation of Proliferation, Wound Healing Properties, Osteogenic and Chondrogenic Potential of Dental Pulp Stem Cells Obtained from Healthy and Periodontitis Affected Teeth

**DOI:** 10.3390/cells10082118

**Published:** 2021-08-18

**Authors:** Hytham N. Fageeh

**Affiliations:** Department of Preventive Dental Science, College of Dentistry, Jazan University, Jazan 45142, Saudi Arabia; hfageeh@jazanu.edu.sa

**Keywords:** dental pulp stem cells, periodontitis, osteogenic, chondrogenic, scaffold, regenerative medicine

## Abstract

Background: Dental pulp tissue within the central cavity of the tooth is composed of dental pulp stem cells (DPSC). These mesenchymal stem cells have good proliferative as well as differentiation potential. DPSC has been isolated even from teeth with inflamed pulps and is found to retain their proliferative and differentiation potential. Little research is available about the viability and differentiation potential of DPSC obtained from teeth with periodontitis. In the present study, the aim was to compare the morphological features, stem cell marker (MSC) expression, proliferation rate, migratory and wound healing properties, osteogenic and chondrogenic differentiation potential of DPSCs obtained from periodontally healthy teeth (hDPSCs) and periodontitis affected teeth (pDPSCs). Methods: Dental pulp tissue was obtained from periodontally healthy volunteers (*n* = 3) and patients with periodontitis undergoing extraction of mobile teeth (*n* = 3). DPSC were isolated using the explant technique and cultured. All the experiments were performed at early passage (Passage 2), late passage (Passage 6) and after cryopreservation. Morphological features of the hDPSCs and pDPSCs were ascertained using microscopy. The expression of cell surface stem cell markers was assessed by the flow cytometry method. The proliferation and growth rate of the cells were assayed by plotting a growth curve from 0–13 days of culture. The migratory characteristics were assessed by wound scratch assay. Osteogenic and chondrogenic differentiation of the cells was assessed using standard protocols with and without induction. Results: DPSCs were successfully obtained from periodontally healthy teeth (hDPSC) and periodontitis-affected teeth (pDPSCs). The data suggests that there were no morphological differences observed in early passage cells between the two cohorts. Cryopreservation did change the morphology of pDSPCs. There was no significant difference in the positive expression of mesenchymal markers CD73, CD90 and CD105 in early passage cells. However, serial passaging and cryopreservation affected the marker expression in pDPSCs. A faint expression of hematopoietic stem cell markers CD34, CD45 and MHC class II antigen HLA-DR was observed in both the cell types. The expression of HLA-DR is upregulated in pDPSCs compared to hDPSC. A significantly slower growth rate and slower wound healing properties was observed in pDPSCs compared to hDPSC. In late passage and after cryopreservation, the migratory ability of pDPSCs was found to be increased drastically. There was no significant difference in osteogenic potential between the two cell types. However, the chondrogenic potential of pDPSCs was significantly lower compared to hDPSc. Yet, pDPSCs showed enhanced osteogenesis and chondrogenesis at late passage as well as after cryopreservation. Conclusion: The results of this novel study shed light on the isolation of viable DPSC from periodontitis-affected teeth. These cells exhibit a slower growth rate and migratory characteristics compared to their healthy counterparts. There was no difference in osteogenic potential but a reduction in chondrogenic potential was seen in pDPSCs compared to hDPSC. The findings reveal that DPSC from periodontitis-affected teeth presents an easy and viable option for regenerative medicine application. Some additional nutritive factors and protocols may be required to attain better regenerative benefits while using pDPSCs.

## 1. Introduction

Stem cells are pluripotent cells endowed with self-renewal, replication and differentiation properties. They can differentiate into several specialized cell types depending on their ambient microenvironment. These cells are found to exist in biological niches or loci within the human body and contribute to repair, healing and normal homeostasis. Stem cells can be isolated from peripheral blood, bone marrow and umbilical cord. The process of harvesting stem cells is fraught with complexity. Collection of stem cells by bone marrow aspiration is painful for the donor and is also associated with significant morbidity. Stem cell harvesting from blood is procedurally complicated and requires a complex armamentarium for the stem cell separation protocol. However, mesenchymal stem cells can be harvested from teeth in a non-invasive manner, making it an easy-access source.

Dental pulp within human teeth is an excellent source of stem cells and is denoted as dental pulp stem cells (DPSCs). These cells were first isolated from the pulp tissues of impacted third molar teeth [1]. DPSCs are believed to be derived from the cranial neural crest and express specific markers such as nestin, S-100 and HNK-1 [2], apart from the routine mesenchymal markers used to characterize them. DPSCs are known to differentiate into several cell types such as neurons, cardiomyocytes, pancreatic islet cells, osteoblasts and chondroblasts [3]. They can be cryopreserved and revived when the necessity arises [4,5]. DPSCs remain quiescent within the dental pulp. Upon isolation and stimulation, they exhibit excellent proliferative properties. These cells also interact well with biomaterials, growth factors and scaffolds which forms the basis for de novo tissue reconstruction [6].

DPSCs can be harvested from teeth with inflamed dental pulps and from teeth with apical periodontitis [7,8,9,10]. Tomasello et al. compared DPSCs and gingival mesenchymal stem cells obtained from periodontally healthy and diseased individuals and found no distinct differences in properties of these cells with regard to proliferation and immunotyping. Interestingly, they found that the stem cells obtained from periodontally diseased individuals demonstrated an overexpression of osteogenic markers [11]. A study by Sun et al. investigated the properties of DPSCs and their association with the nature of teeth from which they are isolated. They demonstrated that teeth with the severe form of aggressive periodontitis also yield DPSCs with reasonable proliferative rates compared to their counterparts isolated from healthy teeth [12]. The authors noted that in an ectopic transplantation model, the DPSCs harvested from teeth with aggressive periodontitis formed dentin-like matrices and pulp-like tissues. However, the potential of tissue formation by these cells was diminished compared to their healthy counterparts. This study was designed to examine periodontitis-affected teeth as a viable source for DPSC. In this study, the effect of periodontitis on pulpal homeostasis was investigated by comparing the affected stem cell population with their healthy counterparts. Furthermore, surface marker characteristics, proliferative ability, osteogenic and chondrogenic potential of DPSCs obtained from periodontitis affected teeth and periodontally healthy teeth was analyzed.

## 2. Materials and Methods

### 2.1. Sample Size Determination and Sample Collection

Written informed consent was obtained from all the volunteers in the study. The Helsinki declaration of good ethical practice in research was adhered to in the design and performance of the present study. The present study was approved by the Scientific Research Committee at the College of Dentistry, Jazan University (CODJU-10245). Dental pulp tissue was obtained from a total of 6 subjects aged between 25 to 35 years. Pulp tissue samples of 3 patients with periodontitis with intact teeth (no caries or exposed pulp) and 3 periodontally healthy subjects were obtained. All subjects were undergoing extractions as a part of routine orthodontic or periodontal therapy. The sample size determination was conducted based on previous studies that have performed similar studies on stem cells [13,14]. However, sample size is not a critical determinant in stem cell experiments as stem cells have uniform properties and are cultured through many passages for the tests. The inclusion criteria for the periodontally healthy subjects were the presence of clinically healthy gingiva and good oral hygiene without calculus, plaque and local factors. Patients with periodontitis were recruited according to the criteria of the latest classification system of periodontal diseases [15]. Based on this, teeth with the presence of abundant local factors such as plaque and calculus and associated with periodontal pockets along with loss of attachment and radiographic evidence of bone loss were chosen for sampling. The exclusion criteria for both the groups were the presence of dental caries in the teeth chosen for sampling, any systemic diseases and debilitating conditions in which dental extraction was contraindicated, and intake of any antibiotic or analgesics for up to 6 months before the study. Pregnant women and lactating mothers were excluded from the study. As described earlier, dental pulp tissue was carefully teased out from the extracted third molar teeth after access opening using a sterile aseptic protocol. The tissues were immediately transferred to the molecular biology laboratory for further processing.

### 2.2. Culture and Expansion of Human Dental Pulp Stem Cells (DPSCs)

Isolation and characterization of DPSCs from the extracted teeth was carried out using the explant culture method [16]. The sampled pulp tissue was minced into tiny fragments. The pieces were placed in 35 mm polystyrene plastic culture dishes. A sufficient amount of fetal bovine serum (FBS) (Gibco, Rockville, MD, USA) was added to the tissues to cover them completely. The tissues were incubated for 24 h at 37 °C and 5% CO_2_. The whole DPSCs culture system was further maintained in DMEM (Invitrogen, Carlsbad, CA, USA). It was supplemented with 20% FBS and antibiotic-antimycotic solution at the same temperature and CO_2_ conditions. The culture medium was replenished twice weekly. The cell growth, health and morphology were monitored regularly with an inverted phase-contrast microscope. After 70–80% confluence was attained, the cells were treated with 0.25% Trypsin-EDTA solution (Invitrogen, Carlsbad, CA, USA) for detachment and transferred to a bigger 25-cm^2^ polystyrene culture flask (Nunc, Rochester, NY, USA). Confluent DPSCs were detached using 0.25% Trypsin-EDTA solution and continuously passaged for expansion. Cells from passages 2 and 6 were used for all experimental assays. DPSCs from patients with periodontitis were denoted as pDPSCs, and cells from periodontally healthy subjects were denoted as hDPSCs.

### 2.3. 3-(4,5-Dimethylthiazol-2-yl)-2,5-diphenyltetrazolium Bromide (MTT) Assay for Metabolic Activity of hDPSCs and pDPSCs

The cells were seeded in 96-well plates (1 × 10^4^ cells per well) and incubated for 24, 48 and 72 h. After incubation, MTT solution (Sigma-Aldrich Corp., St. Louis, MO, USA) at a concentration of 0.5 mg/mL was mixed in each well. The plates were then incubated for 4 h at 37 °C. Post incubation, the medium was removed, and 100 µL dimethyl sulfoxide (DMSO) (Sigma-Aldrich Corp., St. Louis, MO, USA) was added to each well. The absorbance was measured at 570 nm using a Multiskan Spectrum spectrophotometer (Thermo Scientific, San Jose, CA, USA).

### 2.4. Characterization of hDPSCs and pDPSCs by Flow Cytometry

For cell surface marker analysis, the confluent hDPSCs and pDPSCs were trypsinized and washed with PBS twice. The cells were then incubated for 30 min at 4 °C with antihuman-CD73-APC, anti-human-CD90-APC, anti-human-CD105-APC, antihuman-CD34-PE, antihuman-CD45-FITC and anti-human-HLA-DR-APC antibodies (Miltenyi Biotec, Bergisch Gladbach, Germany). Antibody-stained cells were washed twice with PBS. 10,000 cells from each sample were acquired on Attune NxT Flow Cytometer (Thermo Fisher Scientific, Waltham, MA, USA). Isotype controls were used for the detection and to differentiate between positive and negative signals.

### 2.5. Growth Curve Plotting to Assess Proliferative Potential

To examine the proliferative potential of the hDPSCs and pDPSCs, 1 × 10^4^ cells from the second passage were seeded in 12-well cell culture plates. The cell count was estimated every day for 13 days. The growth curve was plotted by analyzing the cell numbers counted over the 13 day period [16].

### 2.6. Analysis of Cell Migration by Wound Scratch Assay

For the measurement of cell migration, confluent hDPSCs and pDPSCs were incubated for 24 h at 37 °C and 5% CO_2_ in a serum-free medium. The cells were mitotically inactivated using 10 µg/mL of mitomycin C (Sigma Aldrich, St. Louis, MO, USA) for 2 h. Wound scratches were created with a sterile plastic 200 μL micropipette tip. After washing, the medium was replaced with a fresh complete growth medium. Photographs of the wounded area were taken at 0 h and 24 h under a microscope. The borders along each wound were marked for evaluation of wound closure. The horizontal distance of migrating cells from the initial wound was measured. The percentage values were derived from the distances measured [17].

### 2.7. Real-Time Quantitative Polymerase Chain Reaction (RT-qPCR) for Quantitative Analysis of Gene Expression Related to Bone and Cartilage Formation

Total RNA was extracted from untreated and treated cells with GeneJet purification columns (Invitrogen, Thermo Scientific, Vilnius, Lithuania). Conversion of one microgram of total RNA into cDNA was conducted with a High-Capacity cDNA Reverse Transcription Kit (Applied Biosystems, Carlsbad, CA, USA). SYBRGreen PCR master mix (Applied Biosystems, Austin, TX, USA) on QuantStudio 5 Real-Time PCR system (Applied Biosystems, Foster City, CA, USA) was used to analyze the expression of genes quantitatively. Primers (IDT, Coralville, IA, USA) such as Runt-related transcription factor 2 (*RUNX2*), osteocalcin (*OCN*), SRY-box transcription factor 9 (*SOX 9*) and aggrecan (*ACAN*) were used for the analysis. The primers used for PCR analysis are listed in Table 1. Expressions of the target genes were normalized to *GAPDH* by the ΔCt technique. mRNA levels were calculated by the ΔΔCt method and were quantified by using the 2^–ΔΔCt^ method.

### 2.8. Osteogenic Differentiation Protocol

The cells were cultured in a 24-well plate (Nunc, Rochester, NY, USA) in a growth medium at a density of 2500 cells per square centimeter. Following 24-h incubation, the growth medium was removed and osteogenic induction medium (DMEM with 1% antibiotic-antimycotic, 0.1 µM of dexamethasone, 50 µM of ascorbate-2-phosphate and 10 mM of β-glycerophosphate (Sigma-Aldrich Corp., St. Louis, MO, USA)) was added. The osteogenic induction medium was changed two times a week. Analysis of osteogenic differentiation was carried out after 7 and 21 days. The treated cells were fixed using 4% paraformaldehyde for osteogenic differentiation analysis. The fixed cells were stained with 2% alizarin red S (pH 4.1–4.3) for twenty minutes. The stained cells were dissolved in 4% NaOH. Quantification of stained osteoblasts was conducted using a spectrophotometer (Multiskan, Thermo Scientific, San Jose, CA, USA) at 450 nm [16].

### 2.9. Chondrogenic Differentiation Protocol

The cells were cultured in a 24-well plate (Nunc, Rochester, NY, USA) in a growth medium at a density of 2500 cells per square centimeter. Following 24-h incubation, the growth medium was removed. DMEM with 1X-ITS, 1 mM of sodium pyruvate, 100 nM of dexamethasone, 50 µg/mL of ascorbate-2-phosphate, 40 µg/mL of L-proline and 10 ng/mL of TGF-β3 (Sigma-Aldrich Corp., St. Louis, MO, USA) was added for chondrogenic induction. The cells were incubated for 28 days at 37 °C in a 5% CO_2_ incubator; the medium was replaced with a fresh medium every 2–3 days. For the control group, the cells were incubated with the plain growth medium. For the analysis of differentiation towards chondrogenic lineage and assessment of glycosaminoglycan (GAGs) content, cells were fixed with 4% paraformaldehyde and stained for glycosaminoglycans using 0.1% toluidine blue. The GAGs were quantified by dissolving stained cells in 4% acetic acid, and the absorbance was measured colorimetrically at 650 nm.

### 2.10. Statistical Analysis

The results were represented as mean ± standard deviation of the values from the three independent experiments. Two experimental groups were compared with each other using the unpaired *t* test (two-tailed). Data were analyzed using GraphPad Prism 8 software (GraphPad Software, La Jolla, CA, USA) for each of the markers utilized. A *p* value < 0.05 was measured as significant (* *p* < 0.05 and ** *p* < 0.01) while a *p* value > 0.05 was interpreted as non-significant.

## 3. Results

### 3.1. hDPSCs and pDPSCs Demonstrated No Significant Differences in Morphology, Metabolic Activity and Mesenchymal Stem Cell (MSC) Marker Expression. pDPSCs Demonstrated Slower Growth at Earlier Passage

**Figure 1:**
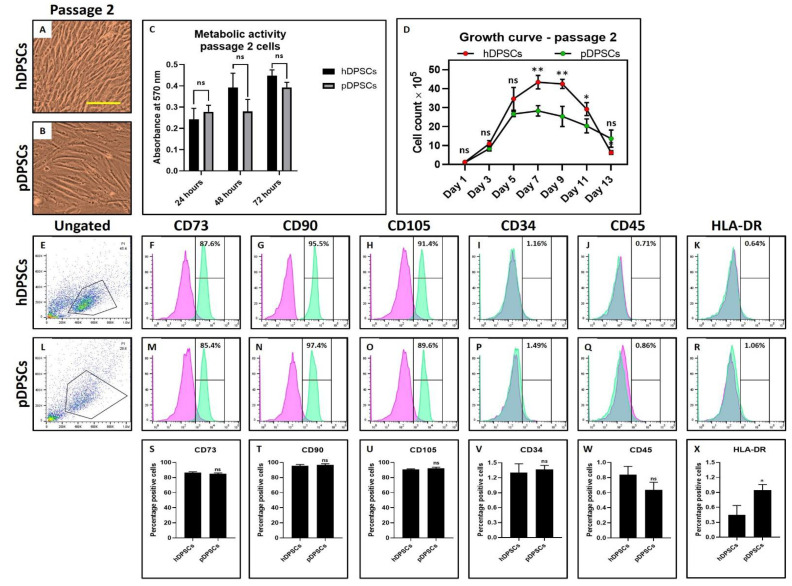
Assessment of morphology, metabolic activity, growth and MSC markers in DPSCs from healthy and periodontitis-affected teeth at Passage 2. (**A**,**B**) photomicrograph showing the morphology of hDPSCs and pDPSCs at Passage 2. Scale bar = 100 μm. (**C**) comparative analysis of the metabolic activity in hDPSCs and pDPSCs at 24, 48 and 72 h. (**D**) comparative analysis of cellular growth in hDPSCs and pDPSCs. (**E**–**X**) comparative analysis of MSC-specific cell surface maker analysis for CD73, CD90, CD105, CD34, CD45 and HLA-DR in hDPSCs and pDPSCs. Experiments were repeated in triplicates for *n* = 3. ns not significant, * *p* < 0.05 and ** *p* < 0.01. hDPSCs: dental pulp stem cells from healthy teeth and pDPSCs: dental pulp stem cells from periodontitis-affected teeth.

Morphological characteristics of hDPSCs and pDPSCs was assessed by observing the cells under a microscope and the expression of MSC-specific cell surface markers by flow cytometry technique. There were no visible changes observed in the MSC-like morphology of DPSCs from both the healthy and periodontitis-affected tissue types (Figure 1A,B). There was no significant difference in the metabolic activity of DPSCs from both tissue types (Figure 1C). Interestingly, a lower proliferation rate in pDPSCs was observed compared to hDPSCs. The number of pDPSCs was significantly lower than hDPSCs at the 7, 9 and 11-day mark (Figure 1D) (*p* < 0.05). Both the cell types demonstrated more than 85% positive expression for CD73, CD90 and CD105 (Figure 1E–H,L–O) and low expression for hematopoietic markers CD34, CD45 and MHC class II antigen HLA-DR (Figure 1I–K,P–R). Both pDPSCs and hDPSCs showed no significant difference in the expression of MSC markers (*p* > 0.05) (Figure 1S–W). However, the expression of HLA-DR expression was significantly higher in pDPSCs as compared to hDPSCs (*p* < 0.05) (Figure 1X).

### 3.2. pDPSCs Demonstrate Slower Migration and Decreased Chondrogenic Potential. No Significant Difference in Osteogenic Potential

**Figure 2:**
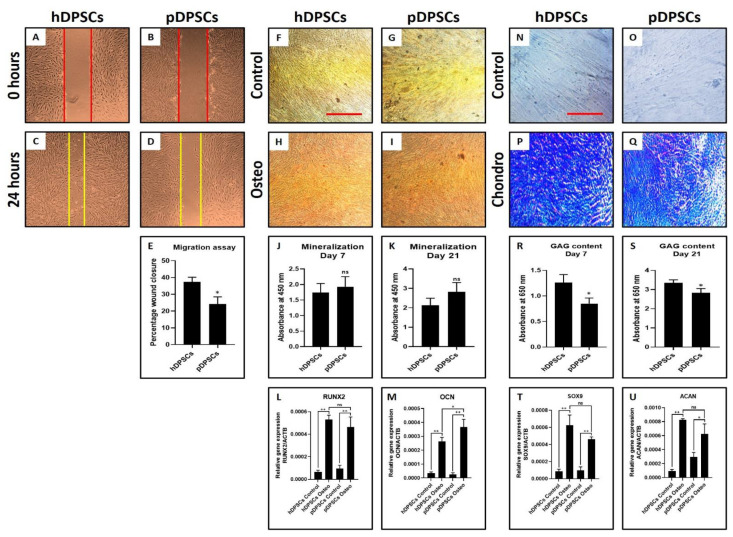
Assessment of migration, osteogenic differentiation and chondrogenic differentiation in DPSCs from healthy and periodontitis-affected teeth at Passage 2. (**A**–**E**) comparative analysis of the migratory ability of hDPSCs and pDPSCs. (**F**–**I**) comparative analysis of osteogenic differentiation in hDPSCs and pDPSCs. (**J**,**K**) comparative analysis of mineralization in osteogenically induced hDPSCs and pDPSCs at day 7 and day 21. (**L**,**M**) comparative analysis of osteogenesis-related genes *RUNX2* and *OCN* in osteogenically induced hDPSCs and pDPSCs at day 21. (**N**–**Q**) comparative analysis of chondrogenic differentiation in hDPSCs and pDPSCs. (**R**,**S**) comparative analysis of GAG content in chondrogenically induced hDPSCs and pDPSCs at day 7 and day 21. (**T**,**U**) comparative analysis of chondrogenesis-related genes *SOX9* and *ACAN* in chondrogenically induced hDPSCs and pDPSCs at day 21. Experiments were repeated in triplicates for *n* = 3. ns: not significant, * *p* < 0.05 and ** *p* < 0.01. hDPSCs: dental pulp stem cells from healthy teeth, pDPSCs: dental pulp stem cells from periodontitis-affected teeth, Osteo: osteogenic induction, *RUNX2*: Runt-related transcription factor 2, *OCN*: osteocalcin, Chondro: chondrogenic induction, GAG: glycosaminoglycan, *SOX9*: SRY-box transcription factor 9 and *ACAN*: aggrecan.

pDPSCs showed reduced migration potential compared to hDPSCs (Figure 2A–E) (*p* < 0.05) as observed from the wound healing assay. Mitotically inactivated hDPSCs migrated more distance than mitotically inactivated pDPSCs (Figure 2C–E). The osteogenic differentiation of hDPSCs and pDPSCs was observed in an induction medium. Both cell types showed comparable osteogenic potential (Figure 2F–K) as observed by alizarin red S staining. Both the cell types showed mineralization in red color (Figure 2H,I) while control groups did not show any retention of red color. However, in comparison of osteogenesis-related genes *RUNX2* and *OCN* by PCR analysis, *OCN* expression was significantly higher in induced pDPSCs than in induced hDPSCs (*p* < 0.05). No difference was noted in the expression of *RUNX2* (*p* > 0.05) (Figure 2L,M). The chondrogenic differentiation of both hDPSCs and pDPSCs was also observed. Toluidine blue specifically stains the sulfated glycosaminoglycans (GAGs) produced by chondrocytes. Staining of GAGs and quantification revealed the chondrogenic potential of both the cells (Figure 2N–S) as observed by dark blue staining in induced groups than uninduced control groups (Figure 2N–Q). GAG content was found to be less in induced pDPSCs compared to induced hDPSCs (*p* < 0.05) (Figure 2R,S). On comparing chondrogenesis-related genes *SOX9* and *ACAN*, *SOX9* expression was significantly lower in induced pDPSCs as compared to induced hDPSCs (*p* < 0.05). There was no significant difference in the expression of *ACAN* (*p* > 0.05) (Figure 2T,U).

### 3.3. Mesenchymal Stem Cell (MSC) Marker Expression Affected in pDPSCs at Late Passage. hDPSCs and pDPSCs Show No Significant Differences in Morphology and Metabolic Activity. pDPSCs Demonstrated Slightly Higher Growth at Late Passage

**Figure 3:**
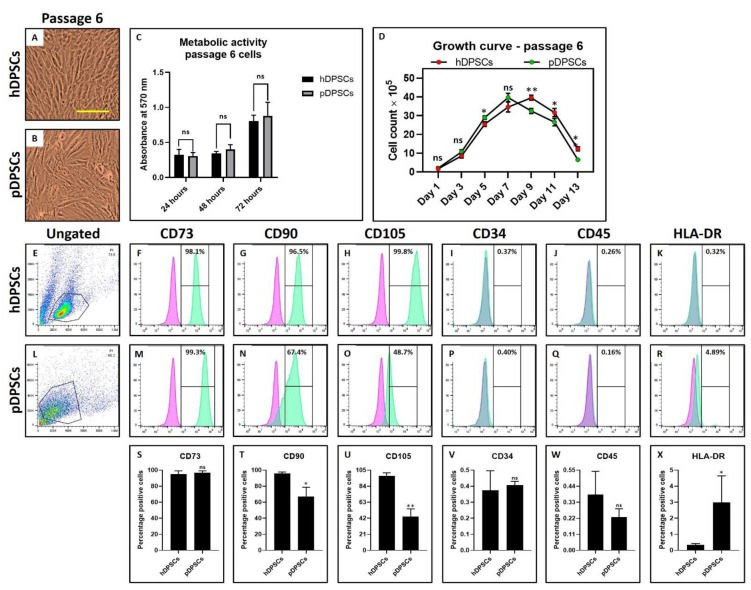
Assessment of morphology, metabolic activity, and MSC markers in DPSCs from healthy and periodontitis-affected teeth at Passage 6. (**A**,**B**) photomicrograph showing the morphology of hDPSCs and pDPSCs at Passage 6. Scale bar = 100 μm. (**C**) comparative analysis of the metabolic activity in hDPSCs and pDPSCs at 24, 48 an 72 h. (**D**) comparative analysis of cellular growth in hDPSCs and pDPSCs. (**E**–**X**) comparative analysis of MSC-specific cell surface maker analysis for CD73, CD90, CD105, CD34, CD45 and HLA-DR in hDPSCs and pDPSCs. Experiments were repeated in triplicates for *n* = 3. ns: not significant, * *p* < 0.05 and ** *p* < 0.01. hDPSCs: dental pulp stem cells from healthy teeth and pDPSCs: dental pulp stem cells from periodontitis-affected teeth.

The morphological characteristics of hDPSCs and pDPSCs was assessed by observing the cells under a microscope and the expression of MSC-specific cell surface markers by flow cytometry technique. No visible changes were seen in the MSC-like morphology of DPSCs from both the healthy and periodontitis-affected tissue types (Figure 3A,B). In both tissue types, there was no significant difference in metabolic activity of DPSCs (Figure 3C). A higher proliferation rate was observed in pDPSCs compared to hDPSCs. The number of pDPSCs was significantly higher than hDPSCs at the day 5 (Figure 3D) (*p* < 0.05). Both the cell types showed no significant difference in the expression of CD73, CD34 and CD45 positive cells (Figure 3E,F,I,J,L,M,P,Q,S,V,W). hDPSCs demonstrated more than 95% positive expression for CD90 and CD105. In contrast, pDPSCs showed a significantly lower expression (Figure 3G,H,N,O,T,U). HLA-DR expression was significantly higher in pDPSCs compared to hDPSCs (Figure 3K,R,X).

### 3.4. pDPSCs Demonstrate Increased Migration, Chondrogenic, and Osteogenic Potential at Late Passage

**Figure 4:**
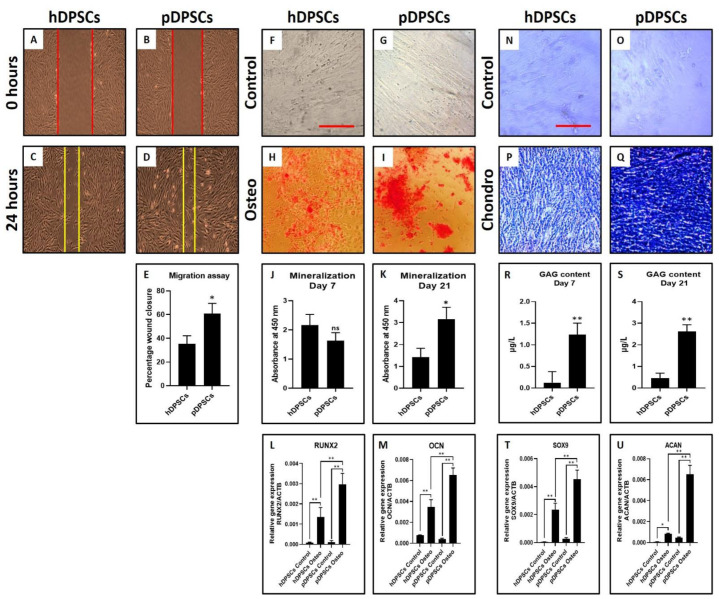
Assessment of migration, osteogenic differentiation and chondrogenic differentiation in DPSCs from healthy and periodontitis-affected teeth at Passage 6. (**A**–**E**) comparative analysis of the migratory ability of hDPSCs and pDPSCs. (**F**–**I**) comparative analysis of osteogenic differentiation in hDPSCs and pDPSCs. (**J**,**K**) comparative analysis of mineralization in osteogenically induced hDPSCs and pDPSCs at day 7 and day 21. (**L**,**M**) comparative analysis of osteogenesis-related genes *RUNX2* and *OCN* in osteogenically induced hDPSCs and pDPSCs at day 21. (**N**–**Q**) comparative analysis of chondrogenic differentiation in hDPSCs and pDPSCs. (**R**,**S**) comparative analysis of GAG content in chondrogenically induced hDPSCs and pDPSCs at day 7 and day 21. (**T**,**U**) comparative analysis of chondrogenesis-related genes *SOX9* and *ACAN* in chondrogenically induced hDPSCs and pDPSCs at day 21. Experiments were repeated in triplicates for *n* = 3. ns: not significant, * *p* < 0.05 and ** *p* < 0.01. hDPSCs: dental pulp stem cells from healthy teeth, pDPSCs: dental pulp stem cells from periodontitis-affected teeth, Osteo: osteogenic induction, *RUNX2*: Runt-related transcription factor 2, *OCN*: osteocalcin, Chondro: chondrogenic induction, GAG: glycosaminoglycan, *SOX9*: SRY-box transcription factor 9 and *ACAN*: aggrecan.

pDPSCs showed enhanced migration potential compared to hDPSCs (Figure 4A–E) (*p* < 0.05) as observed from the wound healing assay. Mitotically inactivated pDPSCs migrated more distance than mitotically inactivated hDPSCs (Figure 4C–E). The osteogenic differentiation of hDPSCs and pDPSCs was observed in an induction medium. pDPSCs showed higher mineralization (Figure 4F–K) as observed by alizarin red S staining. Both the cell types showed mineralization in red color (Figure 4H,I) while control groups did not show any retention of red color. In comparison of osteogenesis-related genes *RUNX2* and *OCN* by PCR analysis, *RUNX2* and *OCN* expression were significantly higher in induced pDPSCs than in induced hDPSCs (*p* < 0.05) (Figure 4L,M). Moreover, the chondrogenic differentiation was observed for both hDPSCs and pDPSCs. Toluidine blue specifically stains the sulfated glycosaminoglycans (GAGs) produced by chondrocytes. Staining of GAGs and quantification revealed the chondrogenic potential of both the cells (Figure 4N–S) as observed by dark blue staining in induced groups than uninduced control groups (Figure 4N–Q). GAG content was found to be more in induced pDPSCs compared to induced hDPSCs (*p* < 0.05) (Figure 4R,S). On comparing chondrogenesis-related genes *SOX9* and *ACAN*, expressions of both genes were significantly higher in induced pDPSCs as compared to induced hDPSCs (*p* < 0.05) (Figure 4T,U).

### 3.5. hDPSCs and pDPSCs Demonstrate Significant Differences in Morphology and Increased Metabolic Activity. Cryopreservation Affected Mesenchymal Stem Cell (MSC) Marker Expression in pDPSCs. pDPSCs Demonstrated Comparable Growth Rate to hDPSCs for Shorter Incubation Time after Cryopreservation

**Figure 5:**
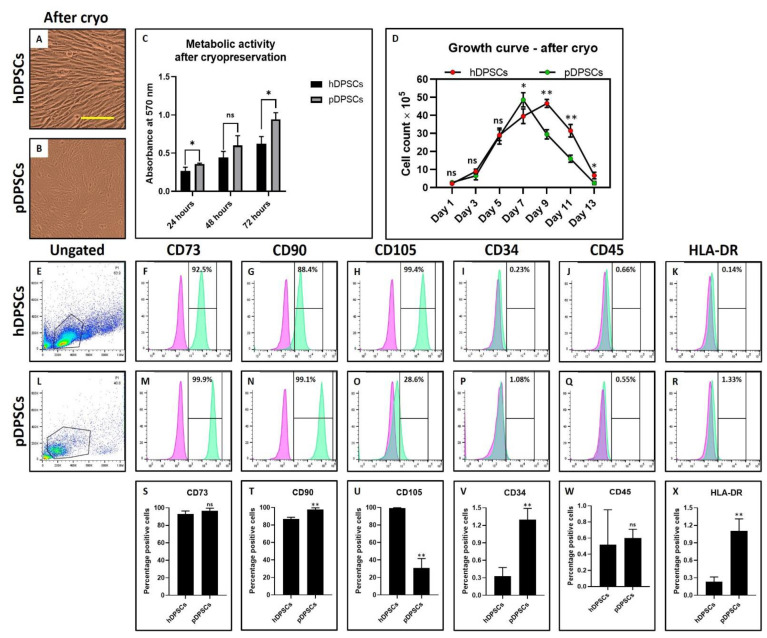
Assessment of morphology, metabolic activity and MSC markers in DPSCs from healthy and periodontitis-affected teeth after cryopreservation. (**A**,**B**) photomicrograph showing the morphology of hDPSCs and pDPSCs at Passage 6. Scale bar = 100 μm. (**C**) comparative analysis of the metabolic activity in hDPSCs and pDPSCs at 24, 48 and 72 h. (**D**) comparative analysis of cellular growth in hDPSCs and pDPSCs. (**E**–**X**) comparative analysis of MSC-specific cell surface maker analysis for CD73, CD90, CD105, CD34, CD45 and HLA-DR in hDPSCs and pDPSCs. Experiments were repeated in triplicates for *n* = 3. ns: not significant, * *p* < 0.05 and ** *p* < 0.01. hDPSCs: dental pulp stem cells from healthy teeth and pDPSCs: dental pulp stem cells from periodontitis-affected teeth.3.6. pDPSCs Demonstrate Increased Migration, Chondrogenic and Osteogenic Potential after Cryopreservation.

The morphological characteristics of hDPSCs and pDPSCs was assessed by observing the cells under a microscope and the expression of MSC-specific cell surface markers by flow cytometry technique. There were visible changes in the MSC-like morphology of pDPSCs (Figure 5A,B). The metabolic activity was significantly increased in pDPSCs as compared to hDPSCs (Figure 5C). A higher proliferation rate was observed in pDPSCs compared to hDPSCs. The number of pDPSCs was significantly higher than hDPSCs at the day 7 (Figure 5D) (*p* < 0.05). Both the cell types showed no significant difference in the expression of CD73 and CD45 positive cells (Figure 5E,F,J,L,M,Q,S,W). hDPSCs demonstrated more than 95% positive expression for CD90 and CD105 (Figure 5G,H,N,T,U). In contrast, pDPSCs showed significantly lower expression for CD105 (Figure 5O,U). CD34 and HLA-DR expression were significantly higher in pDPSCs compared to hDPSCs (Figure 5I,K,P,R,V,X).

**Figure 6:**
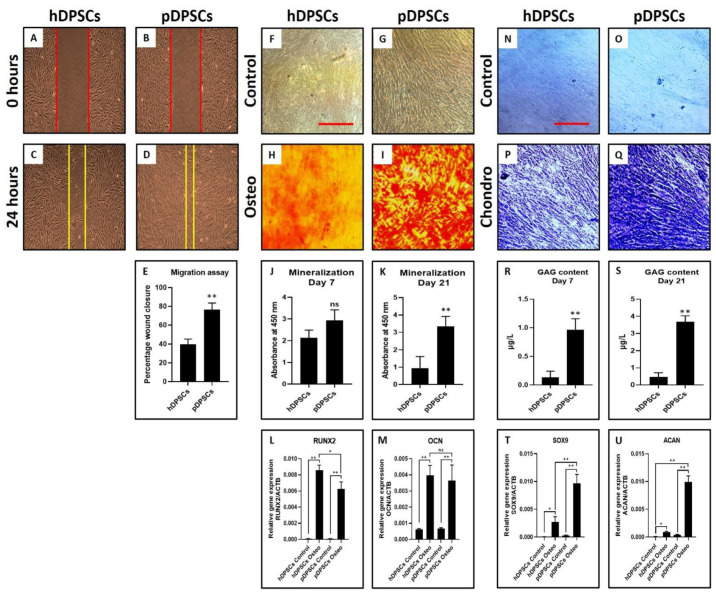
Assessment of migration, osteogenic differentiation and chondrogenic differentiation in DPSCs from healthy and periodontitis-affected teeth after cryopreservation. (**A**–**E**) comparative analysis of the migratory ability of hDPSCs and pDPSCs. (**F**–**I**) comparative analysis of osteogenic differentiation in hDPSCs and pDPSCs. (**J**,**K**) comparative analysis of mineralization in osteogenically induced hDPSCs and pDPSCs at day 7 and day 21. (**L**,**M**) comparative analysis of osteogenesis-related genes *RUNX2* and *OCN* in osteogenically induced hDPSCs and pDPSCs at day 21. (**N**–**Q)** comparative analysis of chondrogenic differentiation in hDPSCs and pDPSCs. (**R**,**S**) comparative analysis of GAG content in chondrogenically induced hDPSCs and pDPSCs at day 7 and day 21. (**T**,**U**) comparative analysis of chondrogenesis-related genes *SOX9* and *ACAN* in chondrogenically induced hDPSCs and pDPSCs at day 21. Experiments were repeated in triplicates for *n* = 3. ns: not significant, * *p* < 0.05 and ** *p* < 0.01. hDPSCs: dental pulp stem cells from healthy teeth, pDPSCs: dental pulp stem cells from periodontitis-affected teeth, Osteo: osteogenic induction, *RUNX2*: Runt-related transcription factor 2, *OCN*: osteocalcin, Chondro: chondrogenic induction, GAG: glycosaminoglycan, *SOX9*: SRY-box transcription factor 9 and *ACAN*: aggrecan.

pDPSCs showed enhanced migration potential compared to hDPSCs (Figure 6A–E) (*p* < 0.05) as observed from the wound healing assay. Mitotically inactivated pDPSCs migrated more distance than mitotically inactivated hDPSCs (Figure 6C–E). The osteogenic differentiation of hDPSCs and pDPSCs was observed in an induction medium. pDPSCs showed higher mineralization (Figure 6F–K) at day 21, as observed by alizarin red S staining. Both the cell types showed mineralization in red color (Figure 6H,I) while control groups did not show any retention of red color. PCR analysis for comparison of osteogenesis-related genes *RUNX2* and *OCN* revealed that *RUNX2* expression was significantly higher in induced hDPSCs than in induced pDPSCs (*p* < 0.05) (Figure 6L,M). However, *OCN* did not show any significant differences in both the cell types. Moreover, the chondrogenic differentiation of both hDPSCs and pDPSCs was observed. Toluidine blue specifically stains the sulfated glycosaminoglycans (GAGs) produced by chondrocytes. Staining of GAGs and quantification revealed the chondrogenic potential of both the cells (Figure 6N–S) as observed by dark blue staining in induced groups than uninduced control groups (Figure 4N–Q). GAG content was found to be higher in induced pDPSCs compared to induced hDPSCs (*p* < 0.05) (Figure 6R,S). Comparison of chondrogenesis-related genes *SOX9* and *ACAN* revealed that expressions of both the genes were significantly higher in induced pDPSCs as compared to induced hDPSCs (*p* < 0.05) (Figure 4T,U).

## 4. Discussion

The principal objective of this study was to assess the properties, proliferative, wound healing, osteogenic differentiation and chondrogenic differentiation potential of DPSC obtained from teeth affected with periodontitis, and compare them to control of periodontally healthy teeth. Periodontitis-affected teeth were investigated if they could be a potential source of DPSC. Teeth with periodontitis show loss of attachment and can become mobile. Consequently, these teeth are associated with questionable prognosis if bone destruction is significant [18]. Such teeth are recommended for extraction as a part of a routine treatment plan [19]. Thus, these teeth can be an easily accessible source for the isolation of DPSC.

DPSCs obtained from healthy teeth (hDPSCs) and periodontitis-affected teeth (pDPSCs) were found to show no significant differences in their morphological characteristics. Additionally, the expression of cell surface markers on both types of DPSC was assessed. Both hDPSCs and pDPSCs showed strong positive expression of the mesenchymal stem cell markers CD73, CD90 and CD105 and faint expression of the hematopoietic stem cell markers CD 34, CD45 and HLA-DR. Although the HLA-DR expression was diminished, it was expressed more in pDPSC compared to hDPSC. Stem cell markers confirm that the cells isolated by our protocol are mesenchymal stem cells. DPSCs are a class/subpopulation of mesenchymal stem cells. Our findings on the cell surface marker expression correlate with previous studies that reported similar results [20,21].

Moreover, the proliferative potential of the stem cells was explored. pDPSC had significantly lower proliferative rates compared to hDPSC at day 7 and day 9 of the 13-day experimental protocol. Also, migratory characteristics of the cells was investigated using the wound scratch assay. Results showed that pDPSC had significantly lower migratory potential relative to hDPSCs. These findings suggest that periodontitis has an impact on DPSC at the molecular level. The periodontium communicates with pulpal tissues through the apical foramen and lateral and accessory canals [22]. In areas of cemental degeneration that occur in periodontitis, the pulp communicates with the periodontium through the patent dentinal tubules [23]. Periodontopathic bacteria aggregate and have been found to colonize the dentinal tubules [24]. These bacteria could affect the pulpal tissue and its homeostasis through the various communication pathways. Fatemi et al. assessed pulp status in 20 periodontitis-affected teeth and found edematous pulps in 58.3% of teeth and fibrotic pulps in 52.1% of the teeth. 43.8% of the teeth exhibited pulp blood vessel dilation and 31.3% of the teeth demonstrated odontoblastic viability [25]. The findings reveal that periodontitis consistently causes inflammation and vascular dilation of the pulp. This phenomenon could result in increased levels of lipopolysaccharide (LPS) of periodontopathic bacteria and proinflammatory cytokines such as IL-1 beta and TNF-alpha in the pulp tissues. Increased cytokines and LPS in the pulp microenvironment could have profound effects on the resident cell populations. It is in this milieu that the DPSCs exist and proliferate. Their proliferative potential and migratory characteristics could be affected compared to their healthy counterparts. These properties could be genotypically and phenotypically preserved in their progeny. Widbiller et al. found that DPSCs show no loss of viability following LPS administration [26]. This finding is in agreement with the present study which found no morphological alterations or loss of viability of both the hDPSCs and pDPSCs. Previously, Liu et al. reported an inhibitory effect of LPS on DPSC proliferation and a stimulatory effect on migration [27]. In the present study, an inhibition in the proliferation and migration of pDPSCs was seen as compared to hDPSCs. The variation in results compared to the previous study [24] could be because of variations in methodology of stem cell isolation, culture protocol and experimental assays performed.

With regard to the effects of passage on cell morphology and MSC marker expression, pDPSc were found to show a change in their properties at a later passage as compared to hDPSc. After Passage 6, the hDPSCs demonstrated more than 95% positive expression for CD90 and CD105. In contrast, pDPSCs overexpressed HLA-DR. A similar effect was observed after cryopreservation where visible changes in the MSC-like morphology of pDPSCs were observed. pDPSCs showed increased metabolic activity. There was a lower expression of CD105 and higher expression of CD34 and HLA-DR.

Our study found no significant differences in osteogenic potential of both the cell types, although the pDPSCs expressed increased levels of the *OCN* gene. This finding is novel and has not been previously reported. Sun et al. reported that the DPSC from periodontitis-affected teeth could form dentin-like matrices and pulp-like tissues upon interaction with scaffolds [12]. Our results show that pDPSCs had a lower chondrogenic potential compared to the hDPSCs which affects glycosaminoglycans production. This finding has not been previously reported in the literature, although it is known that DPSCs have both osteogenic and chondrogenic differentiation potential.

The results of this study suggest that periodontitis-affected teeth can be a viable source for DPSCs. MSCs have extensive physiological effects that make them suitable for therapeutic applications in graft-versus-host disease, acute respiratory syndromes and autoimmune diseases such as multiple sclerosis and Crohn’s disease [28,29,30]. Emerging technologies will help investigators delve deeper into the therapeutic applications of MSCs [31]. Keeping in mind that stem cell experiments are technically difficult to perform and are elaborate in their design, a low sample size is a limitation of the present study. 

Further investigations into cryopreserved DPSCs could establish the effects of supplementing the media. Priming periodontally-affected DPSCs with growth factors, modulating physical and chemical conditions or pharmacological preconditioning with antibiotics could yield stem cells analogous to DPSCs obtained from healthy teeth. Future investigations could focus on the interaction of DPSCs from periodontitis-affected teeth with various scaffolds and bioactive molecules. The paracrine effect of the pDPSCs should be examined for their immunomodulatory effects. Further studies on this topic could lay the foundation for clinical applications of DPSCs in regenerative medicine.

## 5. Conclusions

Earlier studies have confirmed that DPSCs are easy to access and collect. Our findings have provided insights into the isolation of DPSC from periodontitis-affected teeth. Periodontitis is a common oral condition with a significant prevalence rate. Therefore, it is easier to access periodontitis-affected teeth. This study showed that pDPSCs obtained after Passage 6 and revived after cryopreservation had increased migratory characteristics as compared to their unpreserved counterparts from earlier passages. This suggests that DPSCs may be stored in stem cell banks and revived when required representing an advancement in the field of regenerative medicine.

## Figures and Tables

**Table 1:** List of primers used for PCR analysis.

Gene	Forward Primer	Reverse Primer
*RUNX2*	5′-GTG CCT AGG CGC ATT TCA-3′	5′-GCT CTT CTT ACT GAG AGT GGA AGG-3′
*OCN*	5′-GGC GCT ACC TGT ATC AAT GG-3′	5′-TCA GCC AAC TCG TCA CAG TC-3′
*SOX9*	5′-GCC GAA AGC GGG CTC GAA AC-3′	5′-AAA AGT GGG GGC GCT TGC ACC-3′
*ACAN*	5′-GCG AGT TGT CAT GGT CTG AA-3′	5′-TTC TTG GAG AAG GGA GTC CA-3′
*ACTB*	5′-AGA GCT ACG AGC TGC CTG AC-3′	5′-AGC ACT GTG TTG GCG TAC AG-3′

## Data Availability

Data supporting the results of the study are attached in the form of supplementary files.

## References

[1] Gronthos S., Mankani M., Brahim J., Robey P., Shi S. (2000). Postnatal human dental pulp stem cells (DPSCs) in vitro and invivo. Proc. Natl. Acad. Sci. USA.

[2] Almushayt A., Narayanan K., Zaki A.E., George A. (2005). Dentin matrix protein 1 induces cytodifferentiation of dental pulp stem cells into odontoblasts. Gene Ther..

[3] Yu J., He H., Tang C., Zhang G., Li Y., Wang R., Shi J., Jin Y. (2010). Differentiation potential of STRO-1+ dental pulp stem cells changes during cell passaging. BMC Cell Biol..

[4] Lin S.-L., Chang W.-J., Lin C.-Y., Hsieh S.-C., Lee S.-Y., Fan K.-H., Lin C.-T., Huang H.-M. (2014). Static magnetic field increases survival rate of dental pulp stem cells during DMSO-free cryopreservation. Electromagn. Biol. Med..

[5] Gioventù S., Andriolo G., Bonino F., Frasca S., Lazzari L., Montelatici E., Santoro F., Rebulla P. (2012). A novel method for banking dental pulp stem cells. Transfus. Apher. Sci..

[6] Graziano A., D’Aquino R., Laino G., Papaccio G. (2008). Dental pulp stem cells: A promising tool for bone regeneration. Stem Cell Rev..

[7] Alongi D.J., Yamaza T., Song Y., Fouad A.F., Romberg E.E., Shi S., Tuan R.S., Huang G.T.-J. (2010). Stem/progenitor cells from inflamed human dental pulp retain tissue regeneration potential. Regen. Med..

[8] Wang Z., Pan J., Wright J.T., Bencharit S., Zhang S., Everett E.T., Teixeira F.B., Preisser J.S. (2010). Putative Stem Cells in Human Dental Pulp with Irreversible Pulpitis: An Exploratory Study. J. Endod..

[9] Pereira L.O., Rubini M.R., Silva J.R., Oliveira D.M., Silva I.C.R., Poças-Fonseca M.J. (2012). Comparison of stem cell properties of cells isolated from normal and inflamed dental pulps. Int. Endod. J..

[10] Yazid F.B., Gnanasegaran N., Kunasekaran W., Govindasamy V., Musa S. (2014). Comparison of immunodulatory properties of dental pulp stem cells derived from healthy and inflamed teeth. Clin. Oral Investig..

[11] Tomasello L., Mauceri R., Coppola A., Pitrone M., Pizzo G., Campisi G., Pizzolanti G., Giordano C. (2017). Mesenchymal stem cells derived from inflamed dental pulpal and gingival tissue: A potential application for bone formation. Stem Cell Res. Ther..

[12] Sun H.-H., Chen B., Zhu Q.-L., Kong H., Li Q.-H., Gao L.-N., Xiao M., Chen F.-M., Yu Q. (2014). Investigation of dental pulp stem cells isolated from discarded human teeth extracted due to aggressive periodontitis. Biomaterials.

[13] Di Tinco R., Bertani G., Pisciotta A., Bertoni L., Bertacchini J., Colombari B. (2021). Evaluation of Antimicrobial Effect of Air-Polishing Treatments and Their Influence on Human Dental Pulp Stem Cells Seeded on Titanium Disks. Int. J. Mol. Sci..

[14] Park S., Kim J.E., Han J., Jeong S., Lim J.W., Lee M.C., Son H., Kim H.B., Choung Y.-H., Seonwoo H. (2021). 3D-Printed Poly(ε-Caprolactone)/Hydroxyapatite Scaffolds Modified with Alkaline Hydrolysis Enhance Osteogenesis In Vitro. Polymers.

[15] Papapanou P.N., Sanz M., Buduneli N., Dietrich T., Feres M., Fine D.H. (2018). Periodontitis: Consensus report of workgroup 2 of the 2017 World Workshop on the Classification of Periodontal and Peri-Implant Diseases and Conditions. J. Clin. Periodontol..

[16] Patil V.R., Kharat A.H., Kulkarni D.G., Kheur S.M., Bhonde R.R. (2018). Long term explant culture for harvesting homogeneous population of human dental pulp stem cells. Cell Biol. Int..

[17] Cappiello F., Casciaro B., Mangoni M.L. (2018). A Novel In Vitro Wound Healing Assay to Evaluate Cell Migration. J. Vis. Exp..

[18] Kwok V., Caton J.G. (2007). Commentary: Prognosis Revisited: A System for Assigning Periodontal Prognosis. J. Periodontol..

[19] Svärdström G., Wennström J.L. (2000). Periodontal Treatment Decisions for Molars: An Analysis of Influencing Factors and Long-Term Outcome. J. Periodontol..

[20] Rodríguez-Lozano F.J., Bueno C.R., Insausti C.L., Meseguer-Olmo L., Ramírez M.C., Blanquer M.B., Marín N., Martinez S., Moraleda J.M. (2011). Mesenchymal stem cells derived from dental tissues. Int. Endod. J..

[21] Karamzadeh R., Eslaminejad M.B., Aflatoonian R. (2012). Isolation, Characterization and Comparative Differentiation of Human Dental Pulp Stem Cells Derived from Permanent Teeth by Using Two Different Methods. J. Vis. Exp..

[22] Raja V., Emmadi P., Namasivayam A., Thyegarajan R., Rajaraman V. (2008). The periodontal—endodontic continuum: A review. J. Conserv. Dent..

[23] Heasman P.A. (2014). An endodontic conundrum: The association between pulpal infection and periodontal disease. Br. Dent. J..

[24] Adriaens P.A., De Boever J.A., Loesche W.J. (1988). Bacterial Invasion in Root Cementum and Radicular Dentin of Periodontally Diseased Teeth in Humans. J. Periodontol..

[25] Boostani H.R., Fatemi K., Disfani R., Zare R., Moeintaghavi A., Ali S.A. (2012). Influence of moderate to severe chronic periodontitis on dental pulp. J. Indian Soc. Periodontol..

[26] Widbiller M., Eidt A., Wölflick M., Lindner S.R., Schweikl H., Hiller K.-A., Buchalla W., Galler K.M. (2018). Interactive effects of LPS and dentine matrix proteins on human dental pulp stem cells. Int. Endod. J..

[27] Liu Y., Gao Y., Zhan X., Cui L., Xu S., Ma D., Yue J., Wu B., Gao J. (2014). TLR4 Activation by Lipopolysaccharide and Streptococcus mutans Induces Differential Regulation of Proliferation and Migration in Human Dental Pulp Stem Cells. J. Endod..

[28] Petrou P., Gothelf Y., Argov Z., Gotkine M., Levy Y.S., Kassis I. (2016). Safety and Clinical Effects of Mesenchymal Stem Cells Secreting Neurotrophic Factor Transplantation in Patients With Amyotrophic Lateral Sclerosis: Results of Phase 1/2 and 2a Clinical Trials. JAMA Neurol..

[29] Zhao K., Liu Q. (2016). The clinical application of mesenchymal stromal cells in hematopoietic stem cell transplantation. J. Hematol. Oncol..

[30] Martínez-Carrasco R., Sánchez-Abarca L.I., Nieto-Gómez C., Martín García E., Sánchez-Guijo F., Argüeso P. (2019). Subconjunctival injection of mesenchymal stromal cells protects the cornea in an experimental model of GVHD. Ocul Surf..

[31] Zhou T., Yuan Z., Weng J., Pei D., Du X., He C., Lai P. (2021). Challenges and advances in clinical applications of mesenchymal stromal cells. J. Hematol. Oncol..

